# Exploratory radiomics analysis of T1-weighted and FLAIR MRI in relation to Parkinson’s disease motor subtypes

**DOI:** 10.3389/fneur.2026.1920363

**Published:** 2026-09-22

**Authors:** Hyeongjin Lim, Jeongwon Lee, Sangyoon Park, Yongha Gi, Seol-Hee Baek, Byung-Jo Kim, Myonggeun Yoon

**Affiliations:** 1 Department of Biomedical Engineering, Korea University, Seoul, Republic of Korea; 2 FieldCure Co., Ltd., Seoul, Republic of Korea; 3 Department of Neurology, Korea University Ansan Hospital, Korea University College of Medicine, Ansan, Republic of Korea; 4 Department of Neurology, Korea University Anam Hospital, Korea University College of Medicine, Seoul, Republic of Korea

**Keywords:** machine learning, magnetic resonance imaging, motor subtypes, Parkinson’s disease, radiomics

## Abstract

**Background:**

Parkinson’s disease motor subtypes, tremor-dominant (TD) and postural instability/gait difficulty (PIGD), differ in disease trajectory and prognosis, yet their classification depends on MDS-UPDRS-based clinical ratings that are susceptible to inter-rater variability and medication state.

**Purpose:**

This exploratory study investigated whether T1-weighted and FLAIR MRI radiomics show differential associations with TD and PIGD motor subtypes in Parkinson’s disease, and whether the two sequences contribute complementary subtype-relevant information when combined in a multimodal framework.

**Methods:**

Baseline 3 T MRI data from 568 patients with Parkinson’s disease in the PPMI cohort were analyzed (448 TD; 120 PIGD). Radiomics features were extracted from predefined basal ganglia and brainstem regions on T1-weighted and FLAIR images using PyRadiomics. Participants with only T1-weighted or only FLAIR MRI were used as sequence-specific development cohorts; those with both sequences formed a common evaluation cohort. ComBat harmonization was applied within each modality before cohort separation, followed by development-cohort-based feature selection and model fitting. L1-regularized logistic regression models were trained independently for each sequence and evaluated in the common cohort. A stacking model combined T1- and FLAIR-derived probability scores. Model performance was assessed using the area under the precision–recall curve (PR-AUC), balanced accuracy, and F1-score, and Shapley Additive Explanations (SHAP) analysis was used for interpretation.

**Results:**

In the common evaluation cohort (*n* = 148; 118 TD, 30 PIGD), the T1-weighted model achieved a PR-AUC of 0.814, balanced accuracy of 0.829, and F1-score of 0.750. The FLAIR model achieved a PR-AUC of 0.858, balanced accuracy of 0.832, and F1-score of 0.650. The stacked model showed higher performance, with a PR-AUC of 0.928, balanced accuracy of 0.912, and F1-score of 0.818. The T1-weighted model showed relatively better TD classification, whereas the FLAIR model showed higher PIGD sensitivity. SHAP analysis showed that T1-weighted contributions were mainly concentrated in the putamen, while FLAIR contributions were more broadly distributed across basal ganglia and brainstem regions.

**Conclusion:**

T1-weighted and FLAIR MRI radiomics showed distinct subtype-associated error profiles and preliminary evidence of cross-sequence complementarity in this PPMI-based exploratory analysis. These findings require external validation before clinical interpretation, but provide a hypothesis-generating framework for multimodal structural radiomics research in Parkinson’s disease motor subtype characterization.

## 1 Introduction

Parkinson’s disease (PD) exhibits substantial motor heterogeneity, most commonly categorized into tremor-dominant (TD) and postural instability and gait difficulty (PIGD) subtypes based on predominant clinical manifestations (1). These subtypes carry different prognostic implications (2). The TD subtype is generally associated with slower disease progression and relatively preserved functional status, whereas the PIGD subtype exhibits a higher risk of cognitive decline, falls, and rapid functional deterioration (3). Identifying reliable biological correlates of these motor subtypes is therefore of considerable clinical relevance, as it may improve our understanding of phenotypic heterogeneity and support subtype-informed research strategies (4).

Currently, the classification of TD and PIGD subtypes is primarily based on symptom-oriented clinical assessments using the Movement Disorder Society-Unified Parkinson’s Disease Rating Scale (MDS-UPDRS) (5, 6). However, this approach has several limitations. Clinical ratings are inherently susceptible to inter-rater variability and can be influenced by transient symptom fluctuations or medication states (7). More importantly, symptom-based classification may not adequately reflect the underlying pathophysiological differences between motor subtypes (8). Consequently, increasing attention has been directed toward neuroimaging-based approaches as complementary tools for investigating the structural and biological correlates of PD motor subtypes (9).

Among various neuroimaging modalities, structural magnetic resonance imaging (MRI) offers several advantages because it enables noninvasive assessment of brain anatomy and is routinely acquired in clinical settings (10). Previous studies have reported distinct structural alterations between PD motor subtypes, including differences in gray matter atrophy, cortical morphology, and white matter abnormalities (11, 12). In particular, the PIGD subtype has been associated with more pronounced structural changes in regions related to motor and cognitive functions (13). Importantly, different structural MRI modalities provide complementary biological information depending on their imaging contrast (14). T1-weighted (T1w) MRI is particularly effective for evaluating anatomical structures and cortical atrophy, whereas fluid-attenuated inversion recovery (FLAIR) MRI is more sensitive to white matter signal abnormalities and vascular lesions (12, 15). Jointly analyzing T1w and FLAIR may therefore provide a more complete, biologically motivated characterization of subtype-associated structural variation than either sequence alone (14).

Conventional structural MRI studies in PD have largely relied on predefined imaging markers such as regional volume, cortical thickness, or voxel intensity (16). Although informative, these measures may not fully capture complex and subtle tissue heterogeneity embedded within medical images (17). Radiomics-based analysis provides a quantitative framework for extracting a large number of imaging features that describe intensity, shape, and texture characteristics (18, 19). In neurodegenerative disorders, radiomics combined with machine learning has been explored as a method for identifying imaging patterns associated with disease status or clinical phenotypes (20). However, radiomics models can be sensitive to acquisition protocols, scanner differences, preprocessing choices, and harmonization strategies. Therefore, radiomics-based subtype findings require cautious interpretation, particularly given sensitivity to scanner manufacturer differences, acquisition protocols, and harmonization strategy choices, all of which can substantially alter feature distributions even within multicenter datasets such as PPMI.

Several previous studies have applied radiomics approaches to PD diagnosis and motor subtype classification, reporting encouraging classification performance (21, 22). However, these studies often relied on a single imaging modality (21) or provided limited model interpretability (22). In addition, studies that jointly examine T1-weighted and FLAIR MRI radiomics in relation to PD motor subtypes remain limited. Given the potential sensitivity of radiomics features to harmonization specifications, further exploratory studies are needed to evaluate whether subtype-associated radiomics patterns can be observed across structural MRI modalities and how such patterns behave in multimodal modeling frameworks.

The present study was therefore designed as an exploratory radiomics association study using T1-weighted and FLAIR MRI from the PPMI cohort. We hypothesized that radiomics features derived from the two MRI sequences would capture partly distinct and potentially complementary subtype-associated patterns. To test this hypothesis, we pursued three aims: (i) to investigate whether radiomics features derived from T1-weighted and FLAIR MRI are associated with TD and PIGD motor subtype labels; (ii) to evaluate the feasibility of machine learning-based subtype modeling using sequence-specific and multi-sequence radiomics features; and (iii) to characterize the regional and feature-level radiomics patterns contributing to model outputs using SHAP-based interpretation, thereby generating hypotheses for future external validation studies.

## 2 Materials and methods

### 2.1 MRI dataset and participant selection

Structural brain MRI data were obtained from the Parkinson’s Progression Markers Initiative (PPMI) (23), a longitudinal, observational, multicenter study launched in 2010 by The Michael J. Fox Foundation. The PPMI study was approved by the institutional review board at each participating site, and all participants provided written informed consent. For the present exploratory radiomics study, baseline MRI scans were used to maximize sample size while minimizing potential confounding effects of dopaminergic medication exposure. Baseline MRI and clinical data were assessed separately for imaging and clinical eligibility. The initial sequence-specific image pools comprised 491 participants with baseline 3 T T1-weighted MRI and 518 participants with baseline 3 T FLAIR MRI. Image and segmentation quality were visually assessed during the image-processing workflow. No dedicated retrospective motion-correction algorithm was applied. Scans showing substantial image-quality degradation, including motion artifacts, and/or segmentation failure were excluded from further analysis. Motor phenotypes were classified using the MDS-UPDRS-based tremor-dominant/postural instability and gait difficulty (TD/PIGD) ratio described by Stebbins et al. (24). The ratio was calculated by dividing the TD subtype score by the PIGD subtype score. Participants were classified as TD when the ratio was ≥1.15, as PIGD when the ratio was ≤0.90, and as intermediate when the ratio was >0.90 and <1.15. Clinical eligibility for the present TD-versus-PIGD analysis was assessed according to the availability of the MDS-UPDRS information required for subtype classification, motor phenotype, and Hoehn and Yahr stage. In the T1-weighted pool, 66 participants were excluded because the MDS-UPDRS information required for TD/PIGD classification was missing or incomplete, 25 because they met the intermediate motor-phenotype criteria, and 11 because the Hoehn and Yahr stage was unavailable or ≥3. This resulted in 389 participants meeting the clinical eligibility criteria. After intersection with the quality-controlled imaging dataset, 39 clinically eligible participants corresponded to scans that had failed image or segmentation quality control, yielding 350 participants with eligible T1-weighted MRI. In the FLAIR pool, 87 participants were excluded because the MDS-UPDRS information required for TD/PIGD classification was missing or incomplete, 17 because they met the intermediate motor-phenotype criteria, and 19 because the Hoehn and Yahr stage was unavailable or ≥3. This resulted in 395 participants meeting the clinical eligibility criteria. After intersection with the quality-controlled imaging dataset, 29 clinically eligible participants corresponded to scans that had failed image or segmentation quality control, yielding 366 participants with eligible FLAIR MRI. The final sequence-specific datasets partially overlapped: 202 participants had T1-weighted MRI only, 218 had FLAIR MRI only, and 148 had both T1-weighted and FLAIR MRI, resulting in 568 unique participants in the final study cohort. The final cohort comprised 448 participants with TD and 120 with PIGD. Detailed demographic and clinical characteristics are summarized in Table 1, sequence-specific cohort characteristics are provided in Supplementary Table 1, and the available acquisition characteristics are summarized in Supplementary Table 2. All images were resampled to an isotropic resolution of 1 × 1 × 1 mm^3^. Preprocessing included N4 bias field correction, rigid registration to the Montreal Neurological Institute 152 template (25), and skull stripping.

**Table 1:** Demographic and clinical characteristics of the overall Parkinson’s disease cohort stratified by motor subtype.

Variable	TD (*n* = 448 subject)	PIGD (*n* = 120 subject)	*p*-value
Age, years (mean ± SD)	63.6 ± 8.8	65.0 ± 8.6	0.113
Female sex, *n* (%)	165 (36.8)	40 (33.3)	0.548
MoCA	27.0 ± 2.4	27.0 ± 2.4	0.979
MDS-UPDRS-II score	5.7 ± 4.1	8.7 ± 4.1	<0.001
MDS-UPDRS-III score	22.8 ± 9.8	22.4 ± 10.1	0.687
TD subtype score	0.67 ± 0.35	0.18 ± 0.20	<0.001
PIGD subtype score	0.18 ± 0.17	0.49 ± 0.33	<0.001
Hoehn and Yahr stage	1.69 ± 0.46	1.71 ± 0.46	0.659
Side of symptom onset*, *n*/*N* (%)			
Left	194/439 (44.2)	53/117 (45.3)	
Right	241/439 (54.9)	60/117 (51.3)	
Symmetric	4/439 (0.9)	4/117 (3.4)	

Values are presented as mean ± standard deviation or number with percentage. Group differences were assessed between TD and PIGD participants. TD, tremor-dominant; PIGD, postural instability and gait difficulty; SD, standard deviation; MoCA, Montreal Cognitive Assessment; MDS-UPDRS, Movement Disorder Society-Unified Parkinson’s Disease Rating Scale; MDS-UPDRS-II score, Part II total score of the MDS-UPDRS; MDS-UPDRS-III score, Part III total score of the MDS-UPDRS. *Side of symptom onset was available for participants with non-missing records; percentages were calculated based on the corresponding non-missing denominators.

### 2.2 Brain segmentation, Radiomics feature extraction, and WMH analysis

T1-weighted MRI brain structure segmentation was performed using FreeSurfer (26) with default parameters. Regions of interest (ROIs) included the bilateral caudate nucleus, putamen, globus pallidus, midbrain, and pons. For FLAIR MRI, an nnU-Net (27) based segmentation model was trained using 166 PPMI prodromal participants with paired T1-weighted and FLAIR images. These participants were completely independent of the PD participants included in the present radiomics analysis, with no participant-level overlap between the nnU-Net training dataset and either the sequence-specific development cohorts or the common evaluation cohort. Reference labels were generated by registering T1-weighted images to corresponding FLAIR images using Advanced Normalization Tools (ANTs) (28), followed by transformation of FreeSurfer-derived labels into FLAIR space. During reference-label refinement, the transferred anatomical masks were reviewed and manually corrected by three medical imaging researchers with 4, 2, and 3 years of experience in medical image analysis, respectively. The 166 cases were divided into non-overlapping subsets among the three researchers. Because the substantia nigra (SN) was not available as a distinct FreeSurfer label, an additional SN ROI was directly delineated on the FLAIR images during the same reference-label refinement stage. Axial image of SN ROI is provided in Supplementary Figure 1. The refined masks were subsequently reviewed by two senior neurologists with more than 10 and 20 years of clinical experience in neurology, respectively. Their review encompassed the anatomical quality of the T1-to-FLAIR-transferred labels, the appropriateness of manual corrections, the definition of the SN ROI, and the quality of the resulting SN segmentations. Additional corrections were made when necessary. The finalized masks were used as reference labels for nnU-Net training. The trained nnU-Net was then applied to the 366 FLAIR images included in the present radiomics analysis. The resulting segmentation masks underwent final visual quality control by a senior neurologist before radiomics feature extraction. The distribution of ROI sizes in the final FLAIR segmentation masks is summarized in Supplementary Table 3. Radiomics features were extracted from preprocessed MRI images using PyRadiomics (29). For each image, 13 derived filtered images were generated, including gradient, 3D local binary pattern, Laplacian of Gaussian, square, square root, and eight wavelet components. Features were extracted from each predefined ROI on both original and filtered images. For each ROI, 93 texture features were extracted from each filtered image, while 93 texture features and 14 shape features were extracted from the original image. In total, 10,528 features were extracted from T1-weighted MRI and 11,844 features from FLAIR MRI. In addition, white matter hyperintensity (WMH) burden was quantified from native FLAIR images of the 148 participants in the common evaluation cohort using WMH-SynthSeg (30). Total WMH volume was normalized to intracranial volume (ICV) to account for individual differences in head size and was expressed as a percentage of ICV. Differences in normalized WMH burden between the TD and PIGD groups were assessed using the Mann–Whitney U test. The median between-group difference and its 95% confidence interval were estimated using 10,000 bootstrap resamples.

### 2.3 Exploratory radiomics modeling and statistical analysis

The cohort split used for exploratory radiomics modeling is shown in Figure 1a. Participants with T1-weighted MRI only and FLAIR MRI only were used as sequence-specific development cohorts, whereas participants with both sequences were used as a common evaluation cohort for direct comparison of sequence-specific outputs and exploratory assessment of cross-sequence complementarity. Additional undersampling was not applied to enforce equal class proportions within each split, as this would have reduced the available sample size and potentially distorted the clinical distribution represented in PPMI. Instead, the availability-based split was used to preserve the dataset’s intrinsic imaging and clinical structure while enabling exploratory comparison and complementarity assessment between T1-weighted and FLAIR-derived outputs. Because cohort assignment was based on sequence availability, the common evaluation cohort was treated as an internal availability-defined cohort with potential site-, scanner-, and protocol-related distribution differences. Prior to feature selection, we applied the ComBat harmonization (31) method using the neuroCombat package to reduce potential batch effects arising from different scanner models. In the ComBat harmonization model, age and sex were included as covariates to account for biologically relevant variability while adjusting for scanner-related batch effects. This approach aimed to minimize scanner variability while preserving biologically meaningful differences, as in previous studies on PD motor subtype classification (22, 32). Because many scanner types were present only in the common evaluation cohort and were not represented in the sequence-specific development cohorts, ComBat harmonization was performed before cohort splitting within each modality. Scanner type distributions between the sequence-specific development cohort and common evaluation cohort within each modality, as well as the effects of ComBat harmonization, are shown in Supplementary Figures 2, 3, respectively. Subsequent preprocessing and model fitting were performed using only the sequence-specific development cohort. Quantile clipping was applied using the 0.5th and 99.5th percentiles estimated from the development cohort, followed by Yeo–Johnson power transformation (33) with standardization. Highly collinear features were removed using a Pearson correlation threshold of 0.95. Feature selection was performed using L1-regularized logistic regression (34, 35) with class-balanced weighting. When more than 10 features were selected, the 10 features with the largest absolute coefficients were retained. This maximum of 10 features was specified *a priori* as a heuristic constraint to limit model complexity and facilitate feature-level interpretation in this exploratory analysis; it was not selected through data-driven optimization of model performance. A class-balanced L1-regularized logistic regression model was then fitted and applied to the common evaluation cohort. Exploratory model outputs were summarized using the area under the precision–recall curve (PR-AUC), balanced accuracy, and F1-score to consider class imbalance. Ninety-five percent confidence intervals for these performance metrics were estimated using 10,000 bootstrap resamples of the evaluation cohort. These metrics were interpreted as descriptive indices of subtype-related radiomics separability. Shapley Additive Explanations (SHAP) (36, 37) based analyses were used to identify radiomics features contributing to model outputs. Because the final models were linear logistic-regression models, SHA*p* values were calculated analytically in log-odds space using the fitted model coefficients and the mean transformed feature values of the corresponding development cohort as the reference. A *post hoc* stacking-based complementarity analysis was performed using the two sequence-specific probability scores as input variables for a logistic regression meta-model. Stacking was evaluated using stratified five-fold cross-validation. The overall exploratory radiomics modeling workflow is summarized in Figure 1b. Statistical analyses and machine-learning procedures were performed using Python 3.10.20, with NumPy 1.26.4, pandas 2.1.4, SciPy 1.11.4, scikit-learn 1.3.2, and neuroCombat 0.2.12. Additional sensitivity analyses were performed to assess the robustness of the radiomics findings. To examine the potential influence of pre-split harmonization, ComBat was additionally fitted using the development cohort alone and evaluated in common-cohort participants acquired on scanner models sufficiently represented in the development data. Feature-selection stability was assessed by repeating the feature-selection procedure across 1,000 bootstrap resamples of each sequence-specific development cohort and recording the selection frequency of the features retained in the original models. In addition, the FLAIR analysis was repeated after excluding all substantia nigra-derived features to assess the influence of the additional SN ROI on model performance and regional SHAP contribution patterns.

**Figure 1:**
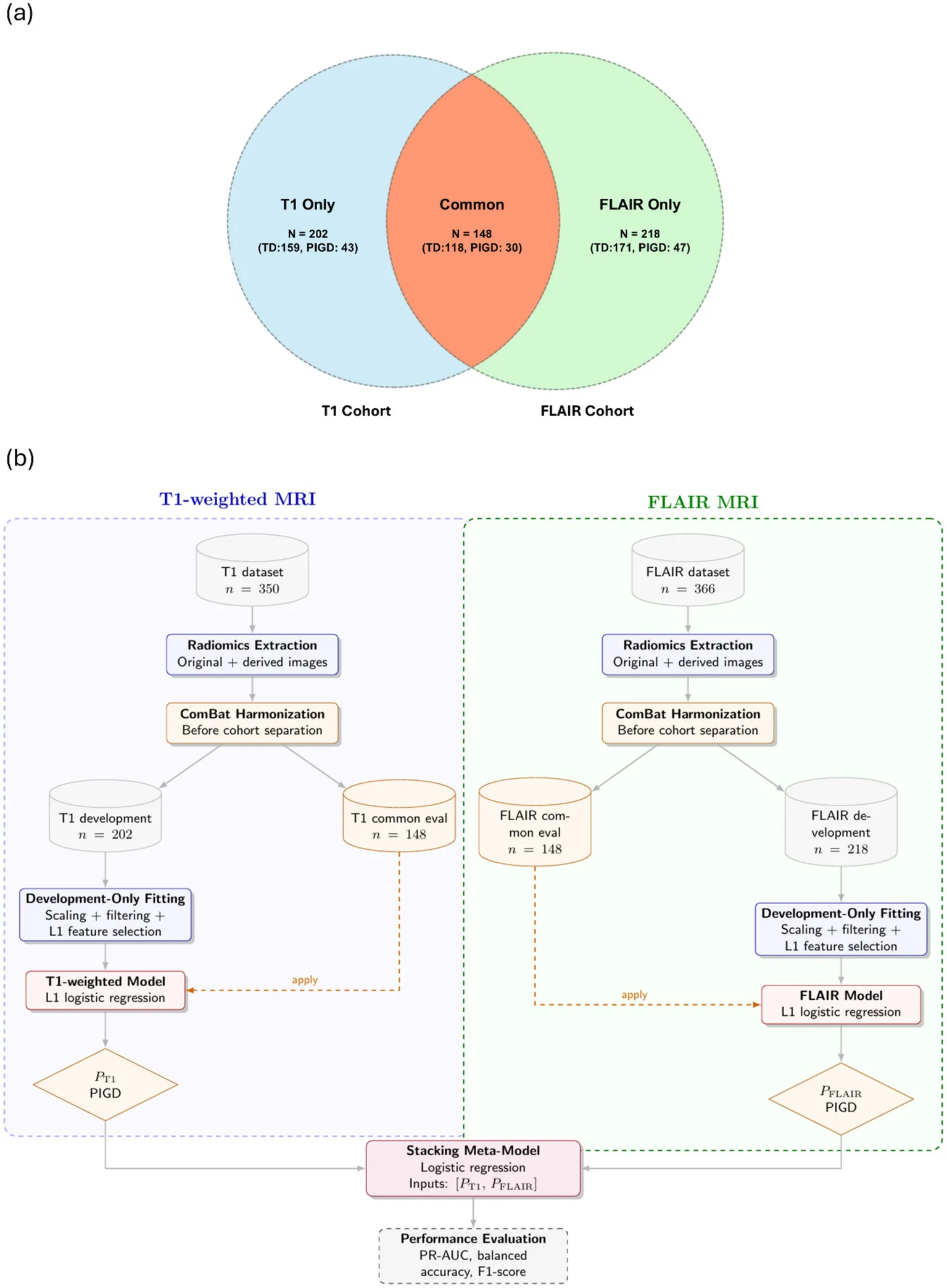
Availability-based cohort composition and exploratory radiomics modeling pipeline. **(a)** Sequence-specific development cohorts and the common evaluation cohort defined by MRI availability. **(b)** Primary radiomics workflow. ComBat harmonization was performed before cohort separation, whereas subsequent preprocessing, feature selection, and model fitting used the development cohorts only. T1-weighted and FLAIR-derived PIGD probabilities were combined using a logistic-regression stacking model with stratified five-fold cross-validation.

## 3 Results

Table 1 shows the demographic and clinical characteristics of the study cohort. The study included 568 participants with Parkinson’s disease, comprising 448 participants with TD and 120 participants with PIGD. Age, sex, Montreal Cognitive Assessment (MoCA) score, MDS-UPDRS-III score, and Hoehn and Yahr stage did not differ significantly between the TD and PIGD groups (*p* > 0.05). In contrast, the PIGD group showed higher MDS-UPDRS-II scores. The TD and PIGD subtype scores also differed markedly between groups, which supports appropriate subtype classification. Supplementary Table 1 shows the characteristics of the sequence-specific development cohorts and the common evaluation cohort. Across the individual cohorts, demographic and general clinical variables did not differ significantly between the TD and PIGD groups (*p* > 0.05). In contrast, MDS-UPDRS-II score, TD subtype score, and PIGD subtype score showed group differences consistent with the overall cohort.

In the common evaluation cohort, the median normalized WMH burden was 0.360% of ICV (IQR, 0.258–0.527%) in the TD group and 0.418% (IQR, 0.266–0.546%) in the PIGD group. The difference was not statistically significant (Mann–Whitney U = 1,591, *p* = 0.395). The median between-group difference was 0.057 percentage points, with a bootstrap 95% confidence interval of −0.075 to 0.166 percentage points, indicating no clear difference in overall WMH burden between the two motor-subtype groups.

To examine the potential influence of the pre-split ComBat harmonization procedure, we performed an additional shared-scanner sensitivity analysis in evaluation subsets restricted to scanner models represented in the corresponding development cohorts. This yielded 30 participants for T1-weighted MRI (26 TD and 4 PIGD) and 62 participants for FLAIR MRI (48 TD and 14 PIGD). Because these shared-scanner subsets contained only 4 PIGD participants for T1-weighted MRI and 14 for FLAIR MRI, the resulting performance estimates were considered descriptive and inconclusive. For T1-weighted MRI, PR-AUC was 0.827 with the original procedure and 0.817 with development-only ComBat, whereas the corresponding FLAIR values were 0.879 and 0.883, respectively. Full results, including balanced accuracy, F1-score, and 95% confidence intervals, are provided in Supplementary Table 4.

Table 2 summarizes the exploratory performance of the T1-weighted, FLAIR, and stacked models in the common evaluation cohort. The T1-weighted model achieved a PR-AUC of 0.814 (95% CI, 0.675–0.924), balanced accuracy of 0.829 (95% CI, 0.739–0.911), and F1-score of 0.750 (95% CI, 0.605–0.854). The FLAIR model achieved a PR-AUC of 0.858 (95% CI, 0.734–0.954), balanced accuracy of 0.832 (95% CI, 0.756–0.899), and F1-score of 0.650 (95% CI, 0.515–0.762). The stacked model showed the highest point estimates, with a PR-AUC of 0.928 (95% CI, 0.850–0.979), balanced accuracy of 0.912 (95% CI, 0.846–0.966), and F1-score of 0.818 (95% CI, 0.704–0.911). In paired bootstrap comparisons, the PR-AUC difference between the stacked and T1-weighted models was 0.113 (95% CI, 0.022–0.226), whereas the difference between the stacked and FLAIR models was 0.070 (95% CI, −0.017–0.170). In the additional repeated cross-validation analysis, the stacking model yielded a mean PR-AUC of 0.926 (95% interval, 0.922–0.930), balanced accuracy of 0.912 (95% interval, 0.899–0.925), and F1-score of 0.821 (95% interval, 0.807–0.835) across 10 repeated stratified five-fold partitions, closely corresponding to the primary five-fold estimates.

**Table 2:** Performance comparison of single-modality and stacking models on the common evaluation cohort.

Model	PR-AUC	Balanced accuracy	F1-score
T1-weighted	0.814 (0.675–0.924)	0.829 (0.739–0.911)	0.750 (0.605–0.854)
FLAIR	0.858 (0.734–0.954)	0.832 (0.756–0.899)	0.650 (0.515–0.762)
Stacking	0.928 (0.850–0.979)	0.912 (0.846–0.966)	0.818 (0.704–0.911)

Ninety-five percent confidence intervals were estimated using 10,000 participant-level bootstrap resamples of the prediction–outcome pairs in the common evaluation cohort. For the stacking model, pooled out-of-fold predictions from stratified five-fold cross-validation were used.

Figure 2 shows the confusion matrices for the sequence-specific and stacked models. The T1-weighted model correctly classified 113 of 118 TD participants and 21 of 30 PIGD participants, whereas the FLAIR model correctly classified 94 of 118 TD participants and 26 of 30 PIGD participants. The stacked model correctly classified 109 of 118 TD participants and 27 of 30 PIGD participants. Figure 3 visualizes the decision boundary of the exploratory stacking model using T1- and FLAIR-derived PIGD probability scores. The two probability scores showed a moderate positive correlation, indicating that the sequence-specific models were related but not redundant. The diagonal decision boundary indicated that both probability scores contributed to the stacked model output. Participants with concordantly low scores were mainly classified as TD, whereas those with concordantly high scores were mainly classified as PIGD.

**Figure 2:**
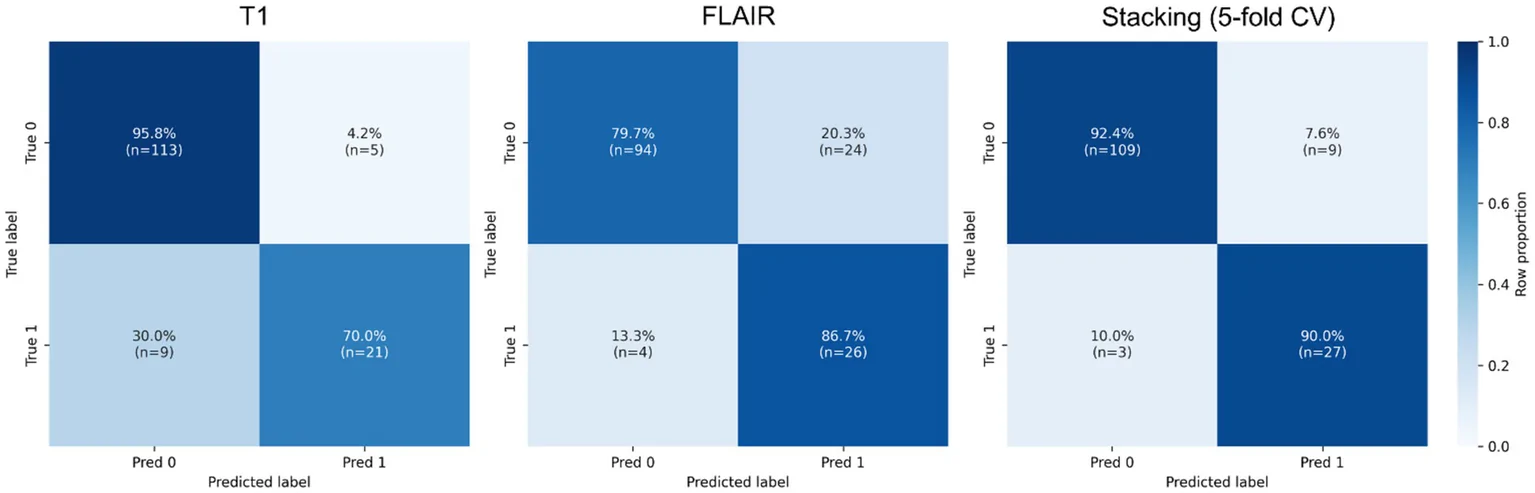
Confusion matrices of the sequence-specific and stacked models in the common evaluation cohort. In all matrices, label 0 denotes TD and label 1 denotes PIGD. Rows indicate the true motor subtype labels, and columns indicate the predicted labels. Each cell reports the row-normalized percentage and the corresponding number of participants. For the stacking model, predictions from the stratified five-fold cross-validation procedure were pooled across folds and summarized as a single aggregated confusion matrix.

**Figure 3:**
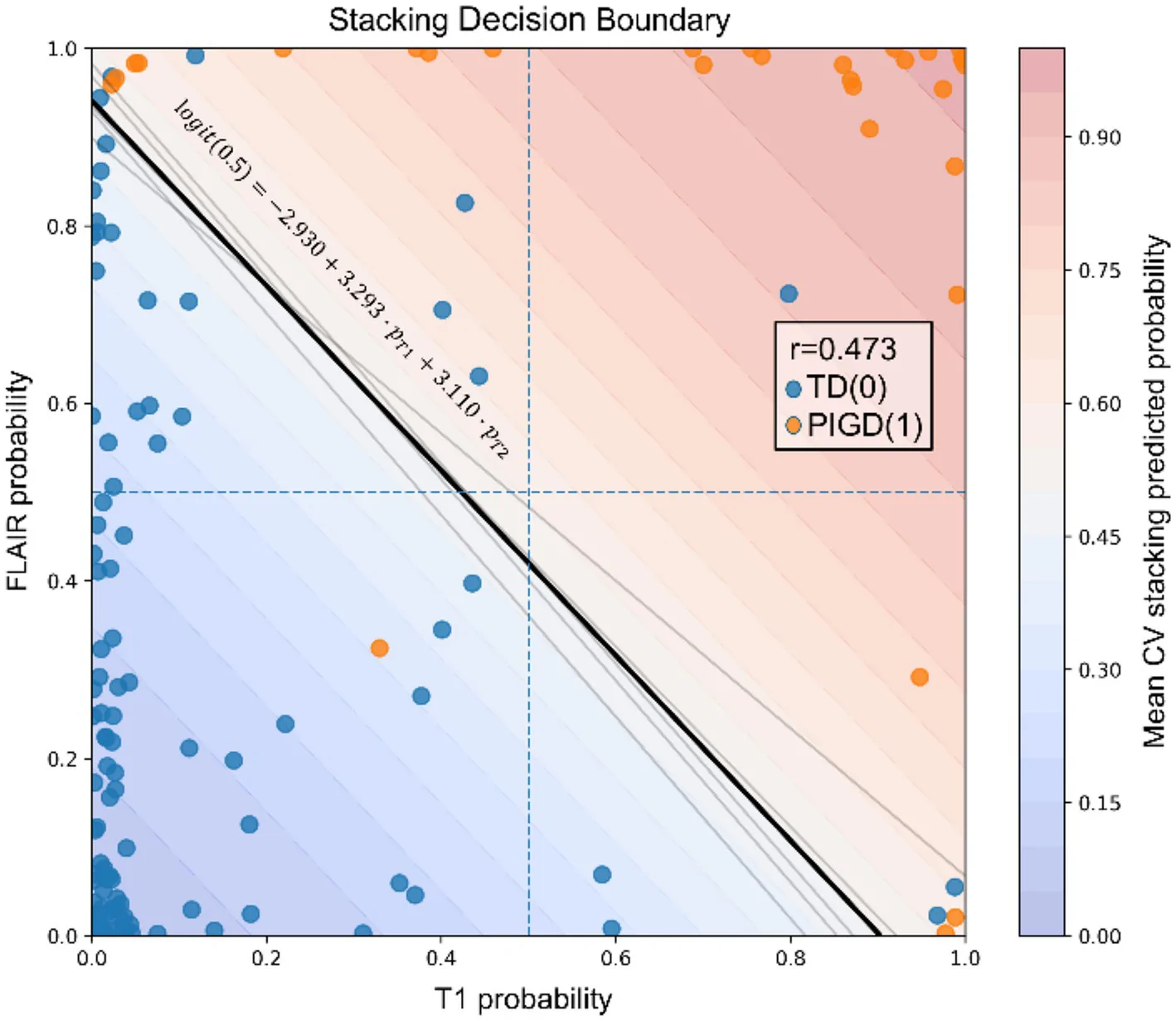
Decision boundary of the exploratory stacking model. Each point represents an individual participant in the common evaluation cohort, plotted according to the PIGD probability scores derived from the T1-weighted and FLAIR models. Point colors indicate the clinically defined motor subtype. The gray lines represent the probability = 0.5 decision boundaries from individual folds of the stratified five-fold cross-validation, and the black line represents the mean decision boundary across folds. The background color indicates the mean predicted probability from the stacking model. The dashed horizontal and vertical lines mark probability values of 0.5 for the FLAIR and T1-weighted models, respectively.

Figure 4 shows the SHAP-based regional contribution maps for the T1-weighted and FLAIR models. In the T1-weighted model, the largest normalized contribution was observed in the left putamen, accounting for 51.4% of the total contribution, followed by the pons, left caudate, left pallidum, and right caudate. In the FLAIR model, the contribution was more widely distributed across the left putamen, pons, left caudate, midbrain, right pallidum, right caudate, and substantia nigra. Figures 5, 6 show the corresponding feature-level SHAP patterns. The T1-weighted model included prominent putamen-derived texture features, whereas the selected FLAIR features were distributed across multiple basal ganglia and brainstem ROIs. Exclusion of all SN-derived features resulted in only modest numerical changes in overall FLAIR performance, with PR-AUC changing from 0.858 to 0.838, balanced accuracy from 0.832 to 0.812, and F1-score from 0.650 to 0.700. As shown in Supplementary Figure 4, regional SHAP attribution was nevertheless redistributed after SN exclusion, with the normalized contribution of the left putamen increasing from 23.1 to 40.7%, followed by the pons, left caudate, right pallidum, and midbrain.

**Figure 4:**
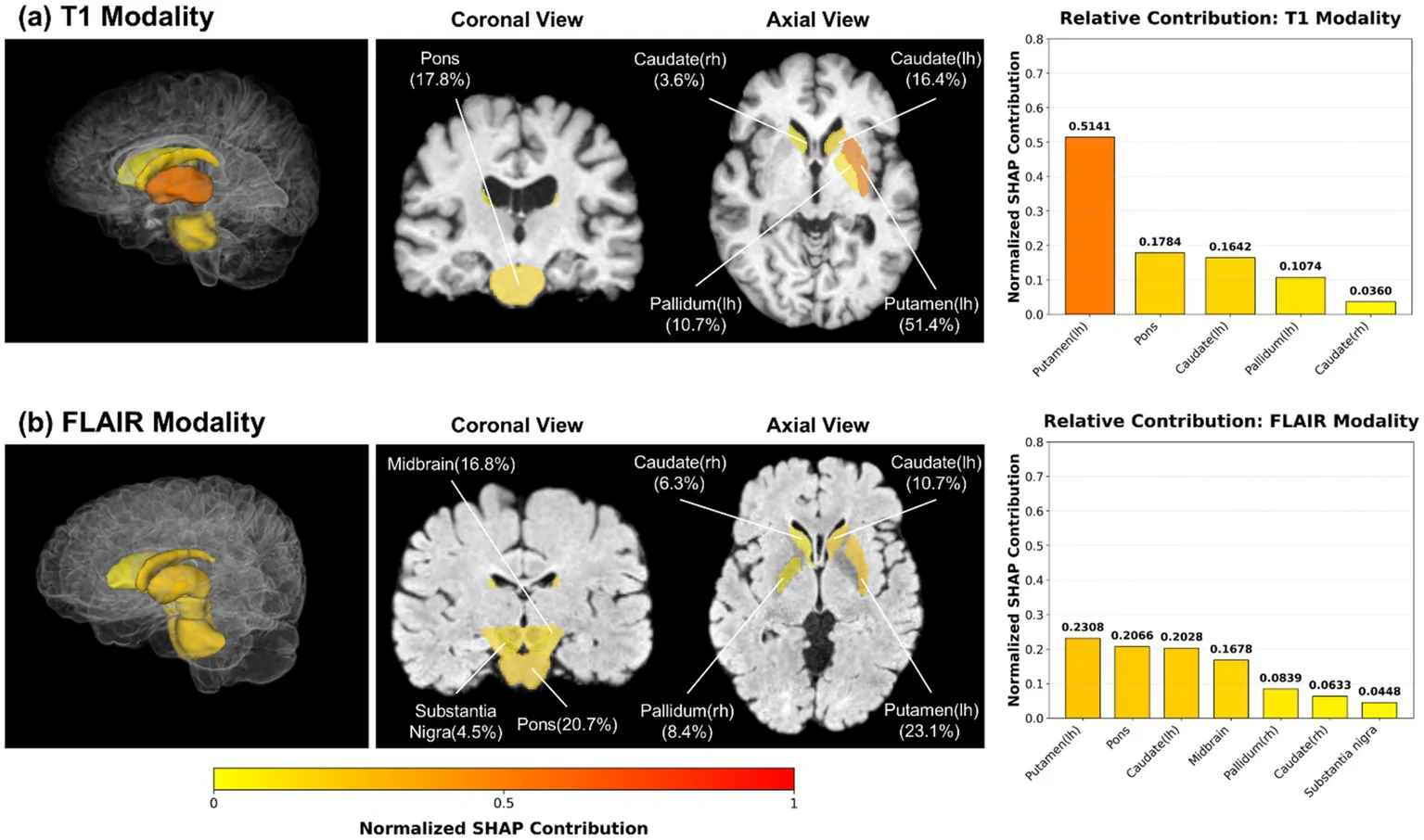
Regional SHAP contribution maps for the T1-weighted and FLAIR models. For each modality, the mean absolute SHAP values of the selected top 10 radiomics features were aggregated according to their corresponding ROIs. The ROI-level contributions were then normalized so that the sum of regional contributions within each modality equaled 1.

**Figure 5:**
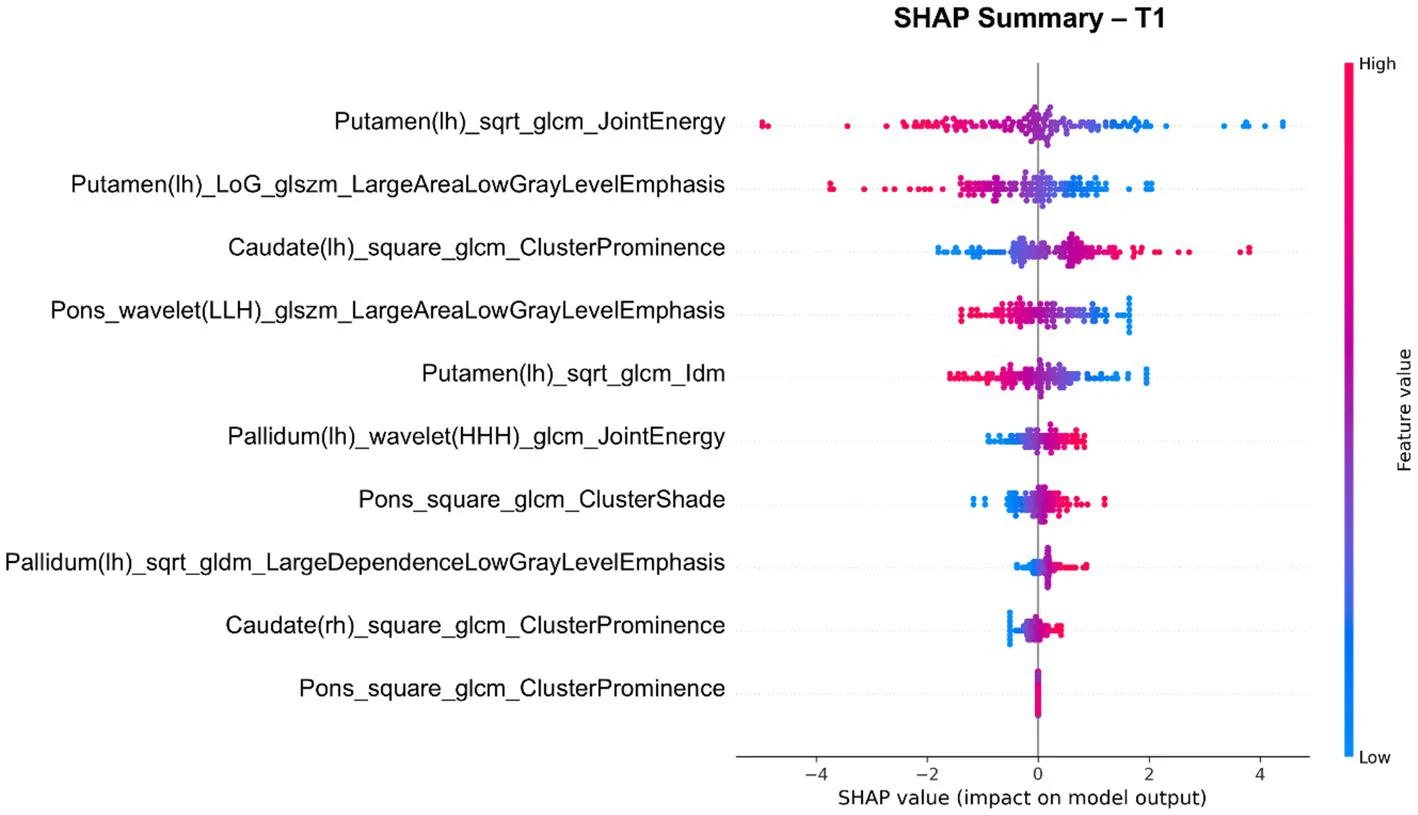
SHAP summary plot of the top selected radiomics features in the T1-weighted model. Each point represents one participant, and the *x*-axis indicates the impact of each feature on model output. Positive SHAP values indicate contribution toward PIGD-oriented prediction.

**Figure 6:**
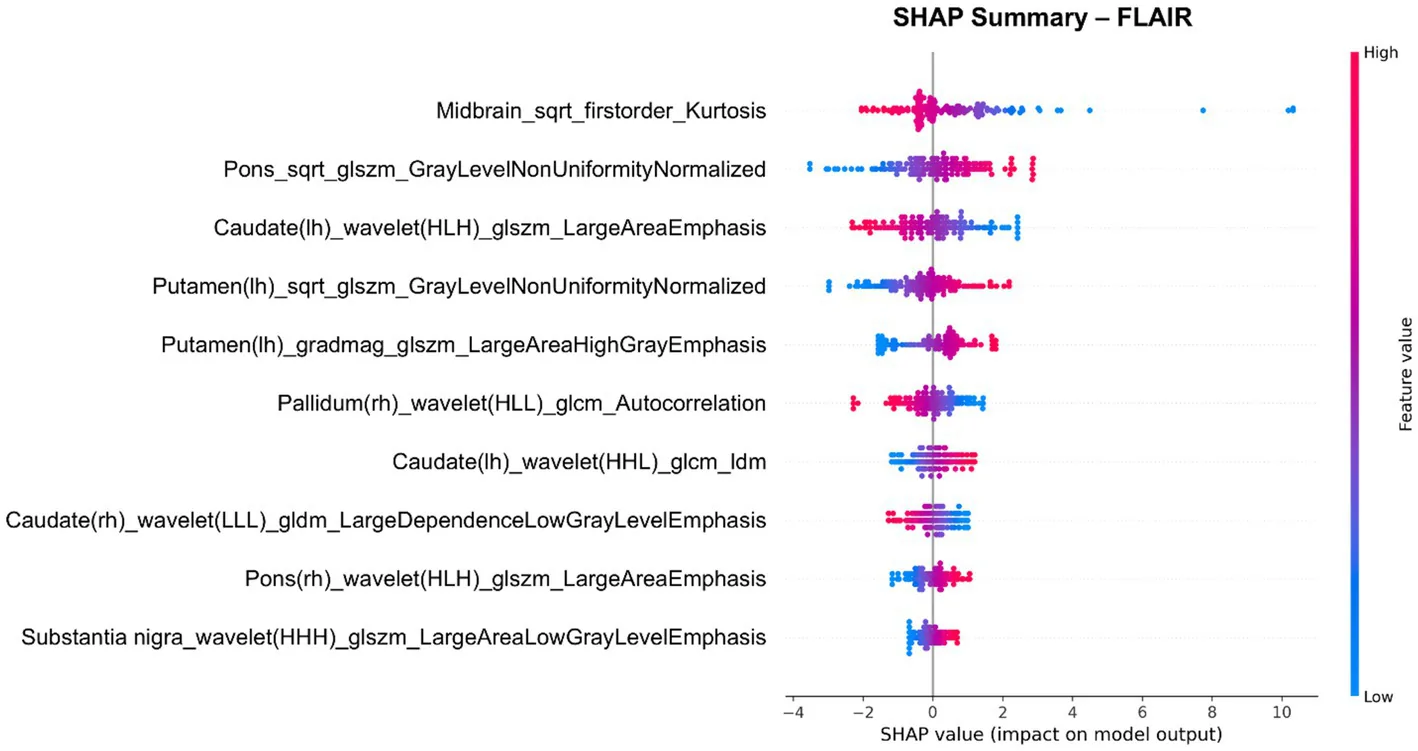
SHAP summary plot of the top selected radiomics features in the FLAIR model. Each point represents one participant, and the *x*-axis indicates the impact of each feature on model output. Positive SHAP values indicate contribution toward PIGD-oriented prediction.

Bootstrap feature-selection analysis demonstrated greater feature-level stability for the T1-weighted model than for the FLAIR model. As detailed in Tables 3, 4, 8 of the 10 features included in the original T1-weighted model were re-selected in more than 50% of 1,000 bootstrap resamples, whereas only 3 of the 10 features included in the original FLAIR model exceeded this threshold. The two most stable T1-weighted features, both derived from the left putamen, were re-selected in all 1,000 bootstrap resamples, while a left pallidal feature was re-selected in 91.2%. In contrast, the three most stable FLAIR features had selection frequencies of 79.1, 77.8, and 62.1%, and the SN-derived feature included in the original FLAIR model was re-selected in 28.2% of bootstrap resamples.

**Table 3:** Bootstrap selection stability of radiomics features included in the original T1-weighted model.

Feature	Selection count (of 1,000)	Selection frequency (%)	Bootstrap selection-frequency rank*
Putamen(lh)_LoG_glszm_LargeAreaLowGrayLevelEmphasis	1,000	100.0	1
Putamen(lh)_sqrt_glcm_JointEnergy	1,000	100.0	2
Pallidum(lh)_sqrt_gldm_LargeDependenceLowGrayLevelEmphasis	912	91.2	3
Putamen(lh)_sqrt_glcm_Idm	832	83.2	4
Caudate(rh)_square_glcm_ClusterProminence	804	80.4	5
Caudate(lh)_square_glcm_ClusterProminence	709	70.9	6
Pons_square_glcm_ClusterShade	692	69.2	7
Pons_wavelet(LLH)_glszm_LargeAreaLowGrayLevelEmphasis	679	67.9	8
Pons_square_glcm_ClusterProminence	477	47.7	9
Pallidum(lh)_wavelet(HHH)_glcm_JointEnergy	221	22.1	13

*Bootstrap selection-frequency rank indicates the feature’s rank among all candidate features according to its bootstrap selection frequency.

**Table 4:** Bootstrap selection stability of radiomics features included in the original FLAIR model.

Feature	Selection count (of 1,000)	Selection frequency (%)	Bootstrap selection-frequency rank*
Putamen(lh)_gradmag_glszm_LargeAreaHighGrayLevelEmphasis	791	79.1	1
Pons_sqrt_glszm_GrayLevelNonUniformityNormalized	778	77.8	2
Midbrain_sqrt_firstorder_Kurtosis	621	62.1	3
Caudate(lh)_wavelet(HLH)_glszm_LargeAreaEmphasis	359	35.9	5
Substantia nigra_wavelet(HHH)_glszm_LargeAreaLowGrayLevelEmphasis	282	28.2	6
Pons_wavelet(HLH)_glszm_LargeAreaEmphasis	163	16.3	8
Pallidum(rh)_wavelet(HLL)_glcm_Autocorrelation	158	15.8	9
Putamen(lh)_sqrt_glszm_GrayLevelNonUniformityNormalized	110	11.0	17
Caudate(rh)_wavelet(LLL)_gldm_LargeDependenceLowGrayLevelEmphasis	110	11.0	18
Caudate(lh)_wavelet(HHL)_glcm_Idm	59	5.9	31

^*^Bootstrap selection-frequency rank indicates the feature’s rank among all candidate features according to its bootstrap selection frequency.

## 4 Discussion

In this exploratory radiomics study, we investigated whether T1-weighted and FLAIR MRI contain subtype-associated imaging information related to tremor-dominant and postural instability/gait difficulty motor subtypes in Parkinson’s disease. Specifically, we evaluated sequence-specific radiomics models, examined whether the two MRI sequences provided complementary information through a stacking analysis, and explored model-derived regional and feature-level patterns using SHAP-based interpretation.

Across the overall cohort and the common evaluation cohort, age, sex, MoCA score, MDS-UPDRS-III score, and Hoehn and Yahr stage were generally comparable between TD and PIGD groups. In contrast, MDS-UPDRS-II scores were significantly higher in the PIGD group. Because MDS-UPDRS-III and Hoehn and Yahr stage did not show significant group differences, the higher MDS-UPDRS-II scores may not simply indicate greater examiner-rated motor severity. Rather, they may reflect greater patient-perceived functional burden in daily activities among participants with the PIGD subtype. This distinction is relevant because the radiomics models should not be interpreted as detecting motor severity itself. Instead, the observed imaging patterns may be associated with subtype-related differences that are linked to functional impact, daily-life disability, or broader motor phenotype expression.

Given the sensitivity of FLAIR MRI to white matter signal abnormalities, we additionally examined global white matter hyperintensity (WMH) burden as a potential contributor to the FLAIR-related findings. No significant difference in normalized WMH burden was observed between the TD and PIGD groups. This finding suggests that a large difference in overall WMH burden is unlikely to account for the observed FLAIR model behavior. However, the analysis does not exclude the possibility of regional, tract-specific, or microstructural white matter alterations that were not captured by the global WMH measure.

The sequence-specific model results suggest that both T1-weighted and FLAIR MRI contained subtype-associated radiomic information, but the two modalities showed different error profiles. The T1-weighted model showed relatively stronger classification of TD participants, whereas the FLAIR model showed higher sensitivity for PIGD participants. This difference suggests that the two sequences may not be redundant. T1-weighted MRI primarily reflects anatomical contrast and structural organization, whereas FLAIR MRI is more sensitive to signal abnormalities and tissue changes that may not be fully captured by T1-weighted imaging. Therefore, the observed difference between T1-weighted and FLAIR model behavior may reflect sequence-dependent sensitivity to different aspects of subtype-associated tissue variation.

The stacking analysis provided further exploratory evidence of cross-sequence complementarity. The stacked model showed the highest point estimates for all three performance measures. Importantly, paired bootstrap analysis supported a higher PR-AUC relative to the T1-weighted model, whereas the corresponding improvement over FLAIR alone was not conclusively demonstrated because the confidence interval for the paired difference included zero. Thus, the stacking findings support potential complementarity but do not establish uniform superiority over both individual sequences. The repeated stratified cross-validation analysis yielded performance estimates closely aligned with the primary five-fold analysis, suggesting that the observed stacking performance was not strongly dependent on a single partition of the common cohort.

The SHAP analysis suggested different regional patterns in the two sequence-specific models. T1-weighted contributions were relatively concentrated in the left putamen, with smaller contributions from the pons, caudate, and pallidum, whereas FLAIR contributions were distributed across a broader set of basal ganglia and brainstem regions. Bootstrap feature-selection analysis, however, showed that the identities of the selected T1-weighted features were more reproducible than those of the FLAIR features: 8 of 10 T1-weighted features, but only 3 of 10 FLAIR features, were re-selected in more than half of the bootstrap samples. This difference indicates that the broader FLAIR feature pattern was more sensitive to resampling of the development cohort and therefore warrants particular caution at the individual-feature level. Because the coefficient-based SHAP formulation used in this study does not explicitly model residual dependence among retained features, SHAP values should likewise be interpreted as descriptive model attributions rather than as unique or causal contributions of individual radiomics features.

The additional SN-exclusion analysis further qualified the cross-sequence SHAP comparison. Removing all SN-derived features produced only modest changes in overall FLAIR classification performance, indicating that the additional SN ROI was not essential for maintaining the observed discriminative performance. However, regional attribution was redistributed after SN exclusion, with the left putamen accounting for a larger proportion of the normalized SHAP contribution. Thus, the broader regional pattern observed in the primary FLAIR model cannot be attributed solely to sequence-specific tissue contrast; it was also influenced to some extent by the difference in ROI composition between the T1-weighted and FLAIR analyses. The cross-sequence SHAP comparison should therefore be considered hypothesis-generating and potentially influenced by both imaging contrast and methodological ROI definition.

The interpretation of the SN findings also requires particular caution. The SN was included as an exploratory FLAIR-based radiomics ROI and was not intended as a dedicated clinical measure of nigral or nigrosome pathology. Conventional FLAIR is not optimized for detailed characterization of nigral iron-related or nigrosome-specific abnormalities, for which susceptibility-sensitive approaches such as SWI or quantitative susceptibility mapping, as well as neuromelanin-sensitive MRI, are more appropriate. In addition, the FLAIR segmentation model relied on reference labels developed from transferred T1-weighted anatomical labels with manual refinement and expert neurologist review. Although the final nnU-Net outputs underwent visual quality control, duplicate independent annotations of the same reference cases were not available and formal inter-rater agreement could therefore not be estimated. Future studies using dedicated nigral imaging and independently replicated manual annotations would provide stronger validation of this ROI.

Among the SHAP-derived patterns, the apparent predominance of left-sided features in the T1-weighted model warrants particularly careful interpretation. Parkinson’s disease often shows asymmetric clinical and neurodegenerative manifestations, and therefore lateralized radiomics patterns may be biologically plausible. However, the present findings do not provide sufficient evidence to conclude that left-sided structures are specifically more involved in PIGD-related imaging changes. In the present analysis, symptom laterality did not show a clear left-dominant pattern. Moreover, the feature-selection procedure may have influenced the apparent lateralization. Because LASSO-based feature selection tends to select one feature among highly correlated predictors, right-sided homologous features may have been excluded when they were strongly correlated with left-sided features. For example, the top T1-weighted putamen feature showed a high left–right correlation of 0.933, suggesting that the selected left-sided feature may partly represent bilateral putaminal texture information. Future studies with larger sample sizes and explicit modeling of symptom laterality are needed to determine whether lateralized radiomics patterns are reproducible and clinically meaningful.

Previous radiomics studies have also explored MRI-based differentiation of TD and PIGD motor phenotypes. Hui et al. applied T1-weighted MRI radiomics of deep brain nuclei to PD motor-subtype classification (21), while Panahi and Hosseini investigated a multimodal T1-weighted and diffusion tensor imaging radiomics approach (22). The present study extends this work by examining T1-weighted and FLAIR MRI, two structural sequences that are commonly available in clinical and multicenter imaging datasets. In addition to evaluating sequence-specific models, we compared their probability outputs within a common cohort and used stacking to explore potential cross-sequence complementarity. SHAP-based regional and feature-level analyses were further used to characterize how the two sequence-specific models derived their outputs. Taken together, this design provides an exploratory framework for examining not only subtype-associated radiomics performance but also the extent to which commonly available structural MRI sequences may provide partially distinct model information.

Despite these exploratory findings and their consistency with prior radiomics research, several limitations should be acknowledged. First, an independent external validation dataset was not available. In addition, the sequence-specific development cohorts and the common evaluation cohort were defined within the PPMI dataset according to MRI sequence availability, rather than through an independent sampling strategy. Because sequence availability may itself have been associated with site-, scanner-, or protocol-specific acquisition practices, cohort membership was not independent of imaging source, and the resulting scanner/source-related distribution differences may have influenced the observed performance estimates. Taken together, the lack of external validation and the availability-based cohort definition limit the generalizability of the present findings. Therefore, the reported performance should be interpreted as an exploratory estimate of subtype-associated radiomic signal within this dataset, not as evidence of generalizable predictive performance. Second, ComBat harmonization was performed before cohort splitting within each modality, because many scanner types in the common evaluation cohort were not represented in the sequence-specific development cohorts. This strategy may have introduced information sharing between cohorts and could therefore lead to optimistic performance estimates. In the shared-scanner sensitivity analysis, development-only ComBat produced numerically similar PR-AUC estimates, particularly for FLAIR; however, this analysis was restricted to substantially smaller subsets and included only four PIGD participants in the T1-weighted comparison. Accordingly, these results are insufficient to meaningfully address the information-sharing concern associated with the primary harmonization strategy. It therefore provides a limited descriptive sensitivity analysis rather than eliminating the information-sharing limitation of the primary analysis. Third, scanner composition was not completely uniform across motor subtypes, as detailed in Supplementary Table 5. Although the scanner–subtype association was not statistically significant in the T1-weighted dataset, a nominal association was observed in FLAIR, and many scanner-specific cells were sparse. ComBat was used to reduce scanner-related feature variation, but harmonization cannot guarantee removal of all confounding associated with nonuniform scanner–subtype distributions. Residual scanner- or protocol-related effects therefore remain possible, particularly for FLAIR. Fourth, the study cohort showed class imbalance, with fewer PIGD than TD participants. Although class-balanced weighting was used during model training and PR-AUC, balanced accuracy, and F1-score were reported, the limited number of PIGD participants may have affected model stability, threshold-dependent performance, and the apparent benefit of stacking. Validation in larger cohorts with adequately powered PIGD samples is therefore required. Finally, the radiomics feature space was high-dimensional relative to the number of participants. The maximum of 10 retained features was specified *a priori* as a heuristic constraint to limit model complexity and facilitate interpretation rather than as an empirically optimized feature number. Alternative feature-number constraints could therefore produce different feature sets or model behavior. More broadly, radiomics features are sensitive to scanner characteristics, acquisition protocols, preprocessing, interpolation, and segmentation quality. These considerations, together with the lower bootstrap feature-selection stability observed for FLAIR, reinforce that the specific selected features and SHAP patterns should be regarded as exploratory and sample-dependent.

In conclusion, T1-weighted and FLAIR MRI radiomics showed subtype-associated information related to TD and PIGD motor phenotypes and exhibited different classification and model-attribution patterns. The stacking analysis suggested potential cross-sequence complementarity, with statistical support for improved PR-AUC relative to T1-weighted MRI but not conclusive evidence of improvement over FLAIR alone. Additional sensitivity analyses provided further context regarding the dependence of the findings on several analytical choices, while also showing that specific feature identities and regional SHAP distributions, particularly for FLAIR, were sensitive to model and ROI specification. These findings remain exploratory and require independent external validation before they can be interpreted as reproducible imaging biomarkers or clinically useful predictors.

## Data Availability

Publicly available datasets were analyzed in this study. This data can be found here: https://www.ppmi-info.org/access-data-specimens/download-data.
