# Supplementary material for: Exploratory radiomics analysis of T1-weighted and FLAIR MRI in relation to Parkinson’s disease motor subtypes
Source: Front Neurol. 2026 Sep 22;17:1920363. doi: 10.3389/fneur.2026.1920363 (PMC13638411; doi:10.3389/fneur.2026.1920363)
Supplement: Supplementary file 1 [file Supplementary_file_1.DOCX]

Supplementary Material

# Supplementary Figures and Tables

**Supplementary Figure 1.** Representative FLAIR-based substantia nigra and midbrain ROI definitions.

**Supplementary Figure 2.** Scanner model distributions in the sequence-specific development and common evaluation cohorts. Distributions are shown separately for the T1-weighted cohorts and the FLAIR cohorts.

**Supplementary Figure 3.** Principal component analysis (PCA) visualization of T1-weighted and FLAIR radiomics features before and after ComBat harmonization. Panels show the feature distributions for (a) the T1-weighted features and (b) the FLAIR features, illustrating the reduction of scanner-related batch effects after harmonization.

**Supplementary Figure 4.** Regional SHAP contribution patterns across FLAIR substantia nigra sensitivity analyses.

## Supplementary Tables

**Supplementary Table 1.** Demographic and clinical characteristics of the T1-weighted development cohort, FLAIR development cohort, and common multi-sequence analysis cohort. Values are presented as mean ± standard deviation or number with percentage. Group differences were assessed between TD and PIGD participants within each cohort. TD, tremor-dominant; PIGD, postural instability and gait difficulty; SD, standard deviation; MoCA, Montreal Cognitive Assessment; MDS-UPDRS, Movement Disorder Society-Unified Parkinson’s Disease Rating Scale.

**Supplementary Table 2.** MRI acquisition characteristics of the T1-weighted and FLAIR scans included in the study.

| **Parameter** | **T1-weighted MRI** | **FLAIR MRI** |
| --- | --- | --- |
| Number of scans | 350 | 366 |
| Field strength | 3.0 T, 350/350 (100%) | 3.0 T, 366/366 (100%) |
| Acquisition plane | Sagittal, 350/350 (100%) | Sagittal, 366/366 (100%) |
| Acquisition type | 3D, 350/350 (100%) | 3D, 366/366 (100%) |
| Slice thickness | 1.0 mm, 350/350 (100%) | 1.0 mm, 366/366 (100%) |
| Sequence description | MPRAGE 280 (80.0%); FSPGR 25 (7.1%); other 3D T1 45 (12.9%) | Multisite 3D FLAIR protocols |
| Weighting | T1 | T2-FLAIR |

**Abbreviations:** TR, repetition time; TE, echo time; TI, inversion time. Detailed scan-level TR, TE, TI, and flip-angle information was not available in the retained imaging metadata and is therefore not reported.

**Supplementary Table 3**. Summary of FLAIR ROI sizes in final segmentation masks (n=366). All images were resampled to 1 × 1 × 1 mm³ isotropic resolution; therefore, voxel counts are numerically equivalent to volumes in mm³.

| **ROI** | **Mean ± SD (voxels)** | **Median** | **Range** |
| --- | --- | --- | --- |
| Right putamen | 4415.2 ± 458.1 | 4408.5 | 3045–5834 |
| Left putamen | 4215.1 ± 427.4 | 4191.0 | 3024–5732 |
| Left globus pallidus | 1904.7 ± 234.6 | 1880.0 | 1287–2548 |
| Right globus pallidus | 1762.5 ± 194.4 | 1754.0 | 1239–2347 |
| Midbrain | 8081.7 ± 816.9 | 8021.0 | 5704–10806 |
| Pons | 16097.0 ± 1950.6 | 15929.0 | 11706–23090 |
| Substantia nigra | 817.9 ± 112.9 | 810.5 | 442–1163 |
| Left caudate | 3715.5 ± 458.3 | 3683.5 | 2663–4968 |
| Right caudate | 3784.0 ± 481.1 | 3743.0 | 2731–5244 |

**Supplementary Table 4**. Shared-scanner sensitivity analysis comparing the primary ComBat approach with development-only harmonization.

**T1, shared-scanner subset: n=30 (26 TD, 4 PIGD)**

| **Harmonization strategy** | **PR-AUC (95% CI)** | **Balanced accuracy (95% CI)** | **F1 (95% CI)** |
| --- | --- | --- | --- |
| Primary all-data ComBat | 0.827 (0.222–1.000) | 0.875 (0.500–1.000) | 0.857 (0–1.000) |
| Development-only ComBat | 0.817 (0.182–1.000) | 0.836 (0.483–1.000) | 0.641 (0–1.000) |

**FLAIR, shared-scanner subset: n=62 (48 TD, 14 PIGD)**

| **Harmonization strategy** | **PR-AUC (95% CI)** | **Balanced accuracy (95% CI)** | **F1 (95% CI)** |
| --- | --- | --- | --- |
| Primary all-data ComBat | 0.879 (0.700–0.986) | 0.902 (0.807–0.971) | 0.783 (0.606–0.919) |
| Development-only ComBat | 0.883 (0.712–0.996) | 0.887 (0.779–0.978) | 0.795 (0.609–0.938) |

**Supplementary Table 5.** Distribution of TD and PIGD motor subtypes across scanner models in the T1-weighted and FLAIR datasets.

**A. T1-weighted MRI**

| **Manufacturer** | **Scanner model** | **TD, n (%)** | **PIGD, n (%)** | **Total, n** |
| --- | --- | --- | --- | --- |
| **SIEMENS** | **TrioTim** | **132 (80.5)** | **32 (19.5)** | **164** |
| **SIEMENS** | **Verio** | **30 (76.9)** | **9 (23.1)** | **39** |
| **GE MEDICAL SYSTEMS** | **SIGNA Architect** | **35 (94.6)** | **2 (5.4)** | **37** |
| **SIEMENS** | **Skyra** | **18 (78.3)** | **5 (21.7)** | **23** |
| **Philips** | **Ingenia** | **14 (87.5)** | **2 (12.5)** | **16** |
| **SIEMENS** | **Prisma** | **9 (60.0)** | **6 (40.0)** | **15** |
| **SIEMENS** | **Prisma_fit** | **11 (84.6)** | **2 (15.4)** | **13** |
| **GE MEDICAL SYSTEMS** | **DISCOVERY MR750** | **8 (72.7)** | **3 (27.3)** | **11** |
| **Philips Medical Systems** | **Achieva dStream** | **5 (62.5)** | **3 (37.5)** | **8** |
| **SIEMENS** | **Biograph_mMR** | **2 (40.0)** | **3 (60.0)** | **5** |
| **Philips Medical Systems** | **Achieva** | **3 (60.0)** | **2 (40.0)** | **5** |
| **Siemens Healthineers** | **MAGNETOM Vida** | **3 (60.0)** | **2 (40.0)** | **5** |
| **Philips Medical Systems** | **Ingenia** | **2 (66.7)** | **1 (33.3)** | **3** |
| **Siemens** | **MAGNETOM Vida** | **1 (50.0)** | **1 (50.0)** | **2** |
| **GE MEDICAL SYSTEMS** | **DISCOVERY MR750w** | **1 (100.0)** | **0 (0.0)** | **1** |
| **Philips** | **MR 7700** | **1 (100.0)** | **0 (0.0)** | **1** |
| **Siemens Healthineers** | **MAGNETOM Prisma** | **1 (100.0)** | **0 (0.0)** | **1** |
| **Siemens Healthineers** | **MAGNETOM Skyra** | **1 (100.0)** | **0 (0.0)** | **1** |
| **Total** |  | **277 (79.1)** | **73 (20.9)** | **350** |

**B. FLAIR MRI**

| **Manufacturer** | **Scanner model** | **TD, n (%)** | **PIGD, n (%)** | **Total, n** |
| --- | --- | --- | --- | --- |
| **SIEMENS** | **Prisma_fit** | **87 (81.3)** | **20 (18.7)** | **107** |
| **SIEMENS** | **Skyra** | **52 (74.3)** | **18 (25.7)** | **70** |
| **SIEMENS** | **Prisma** | **25 (61.0)** | **16 (39.0)** | **41** |
| **GE MEDICAL SYSTEMS** | **SIGNA Architect** | **33 (94.3)** | **2 (5.7)** | **35** |
| **SIEMENS** | **Verio** | **26 (78.8)** | **7 (21.2)** | **33** |
| **Philips** | **Ingenia** | **13 (86.7)** | **2 (13.3)** | **15** |
| **Siemens Healthineers** | **MAGNETOM Prisma Fit** | **15 (100.0)** | **0 (0.0)** | **15** |
| **GE MEDICAL SYSTEMS** | **DISCOVERY MR750** | **7 (70.0)** | **3 (30.0)** | **10** |
| **Siemens Healthineers** | **MAGNETOM Skyra** | **8 (100.0)** | **0 (0.0)** | **8** |
| **Philips Medical Systems** | **Achieva dStream** | **4 (57.1)** | **3 (42.9)** | **7** |
| **Philips Medical Systems** | **Achieva** | **3 (60.0)** | **2 (40.0)** | **5** |
| **Siemens Healthineers** | **MAGNETOM Prisma** | **5 (100.0)** | **0 (0.0)** | **5** |
| **Siemens Healthineers** | **MAGNETOM Vida** | **3 (60.0)** | **2 (40.0)** | **5** |
| **Philips Medical Systems** | **Ingenia** | **2 (66.7)** | **1 (33.3)** | **3** |
| **SIEMENS** | **TrioTim** | **3 (100.0)** | **0 (0.0)** | **3** |
| **Siemens** | **MAGNETOM Vida** | **1 (50.0)** | **1 (50.0)** | **2** |
| **GE MEDICAL SYSTEMS** | **DISCOVERY MR750w** | **1 (100.0)** | **0 (0.0)** | **1** |
| **Philips** | **MR 7700** | **1 (100.0)** | **0 (0.0)** | **1** |
| **Total** |  | **289 (79.0)** | **77 (21.0)** | **366** |

**C. Association between scanner model and motor subtype**

| **Modality** | **N (TD/PIGD)** | **χ²** | **df** | **P value** | **Cramér's V** | **Expected cells <5** |
| --- | --- | --- | --- | --- | --- | --- |
| **T1-weighted** | **350 (277/73)** | **20.745** | **17** | **0.238** | **0.243** | **66.7%** |
| **FLAIR** | **366 (289/77)** | **29.474** | **17** | **0.030** | **0.284** | **58.3%** |
